# Do Islanders Have a More Reactive Behavioral Immune System? Social Cognitions and Preferred Interpersonal Distances During the COVID-19 Pandemic

**DOI:** 10.3389/fpsyg.2021.647586

**Published:** 2021-04-30

**Authors:** Ivana Hromatko, Andrea Grus, Gabrijela Kolđeraj

**Affiliations:** Department of Psychology, Faculty of Humanities and Social Sciences, University of Zagreb, Zagreb, Croatia

**Keywords:** behavioral immune system, social cognitions, interpersonal distance, COVID-19, xenophobia

## Abstract

Insular populations have traditionally drawn a lot of attention from epidemiologists as they provide important insights regarding transmission of infectious diseases and propagation of epidemics. There are numerous historical instances where isolated populations showed high morbidity once a new virus entered the population. Building upon that and recent findings that the activation of the behavioral immune system (BIS) depends both upon one’s vulnerability and environmental context, we predicted that, during the COVID-19 pandemic, place of residence (island vs. mainland) explains a significant proportion of variance in preferred interpersonal distances, animosity toward strangers, and willingness to punish those who do not adhere to COVID-19 preventive measures. With 48 populated islands, Croatia provides a fruitful testing ground for this prediction. We also opted to explore relations among BIS-related variables (pathogen disgust, germ aversion, and perceived infectability) and social cognitions in a more natural context than has previously been done. The study was conducted online, on Croatian residents, during April and May 2020. As expected, the BIS variables contributed significantly to preferred interpersonal distances, negative emotions toward strangers, and willingness to punish those who do not adhere to COVID-19 preventive measures. Furthermore, our results showed that geographical location explained a significant amount of variance in preferred social (but not personal and intimate) distances and negative emotions toward foreigners. As Croatian islands are extremely frequent travel destinations, these differences between mainlanders and islanders cannot be explained by the lack of exposure to foreigners. Additionally, we found that scores on preferred interpersonal distances, pathogen disgust, and germ aversion were significantly higher compared to those obtained in Croatian samples before the COVID-19 pandemic. Furthermore, men scored higher in perceived infectability than before the COVID-19 pandemic, and women did not, which reflects the objectively higher risk of SARS-CoV-2 for men than for women. Taken together, our results support the notion that BIS is a highly adaptive and context-dependent response system, likely more reactive in more susceptible individuals.

## Introduction

Epidemics are not a novelty in human evolutionary history. In fact, they have plagued humanity from the very beginnings. Even the recent pandemic caused by the novel coronavirus SARS-CoV-2, leading to deaths of more than 2.7 million people as of March 31, 2021 per the WHO, is not an unprecedented event in human history. Having in mind that humans evolved alongside numerous pathogens, it comes as no surprise that there exists a unique system composed of various cognitive and affective processes and behaviors whose main goal is to protect the organism from coming into contact with the infectious disease in the first place. The behavioral immune system (BIS), as defined by Schaller (2006), has a unique role in shaping a variety of human behaviors, from basic avoidance of rotten food to social cognitions. Disgust, the emotion with a central role in this system, serves as a main motivator toward pathogen and disease avoidance. In other words, the higher the disgust sensitivity or disgust elicited, the higher the motivation to implement stimuli avoiding behaviors (Curtis and Biran, 2001; Oaten et al., 2009).

While BIS activation is closely related to disgust, it also depends on the context and various situational cues that can make disease threats more salient (Schaller, 2016), therefore making behaviors that are a product of BIS activation more pronounced. Indeed, recent findings corroborate the notion that some sort of sensitization to pathogen threat occurred during this global health crisis, resulting in heightened scores on BIS-related traits on a group level (Miłkowska et al., 2021; Stevenson et al., 2021). It should be noted that some authors cautioned against superficial application of BIS theoretical framework in research of psychological processes during a pandemic (Ackerman et al., 2020) and others further elaborated on mismatches between ancestral environments in which the adaptations collectively called BIS may have evolved and contemporary conditions of living (Ackerman et al., 2020; Varella et al., 2021). However, even though sickness cues are not easily detectable in early phases of SARS-CoV-2 infection, especially in asymptomatic cases, contemporary humans collect and process disease-threat relevant information through various channels. The daily updated numbers of new cases and deaths, as well as news about available interventions such as the development of treatment protocols, drugs, and vaccines, are easily accessible on various media platforms; in fact, there is a surplus of information, not a lack thereof, and the resulting *infodemic* has already been associated with a rise in health-related anxiety disorders (Jokic-Begic et al., 2020). In this sense, the COVID-19 pandemic provides a unique opportunity to study BIS in more naturalistic circumstances, as compared to inducing disease salience and pathogen threat artificially via priming.

The last serious outbreak, although much smaller in size than the current one, the Ebola outbreak in 2014, showed that certain social cognitions can become more pronounced during times of disease threat. Kim et al. (2016) showed that, when disease threat is especially salient and when people feel fearful and vulnerable to disease, they also express more xenophobic attitudes. A similar pattern has been observed during this pandemic: people tend to express more negative attitudes toward foreigners (Sorokowski et al., 2020) and increased support for conservative political candidates (Karwowski et al., 2020). In our evolutionary history, outgroup members might have posed a threat because they could have been carriers of some new, previously unencountered pathogen, thus increasing the risk of disease for the in-group. Additionally, outgroup members possibly don’t know or understand customs that could have been set in place to minimize the risk of disease spread, thus once again increasing the risk of pathogen transmission for the in-group (Schaller, 2016). Of course, xenophobia directed against group identity markers is unlikely to prevent disease transmission in modern contexts, making group identity only a weak correlate of infection risk (Ackerman et al., 2020). Indeed, it seems that resistance to foreign norms, rather than avoidance of novel pathogens, better explains the relationship between pathogen avoidance and outgroup prejudice (Karinen et al., 2019). However, there is ample evidence that disease and pathogen salience had a role in shaping various cultural specificities (Schaller and Murray, 2010). Cultures that have historically been more exposed to various infectious diseases have more xenophobic and conservative attitudes (Fincher et al., 2008; Murray and Schaller, 2010; Terrizzi et al., 2013). A large cross-cultural study (Tybur et al., 2016) recently showed that national parasite stress relates to traditionalism, defined as an aspect of conservatism especially related to adherence to group norms, but not to social dominance orientation, which is an aspect of conservatism especially related to endorsements of intergroup barriers and negativity toward ethnic and racial outgroups. Schaller and Murray (2008) showed that the association between disease prevalence and regional variability in extraversion, openness to experience and (female) sociosexuality remained significant even after controlling for variety of other variables (e.g., latitude, temperature, life expectancy, and GDP per capita) which might influence these cultural variations.

On the individual level, exposure to a disease prime can lead participants with high perceived vulnerability to disease to rate themselves as less agreeable and less open to experience, facilitate avoidant tendencies (Mortensen et al., 2010), increase ethnocentric attitudes (Navarrete and Fessler, 2006), as well as conformity (Wu and Chang, 2012). Furthermore, sensitivity of the BIS, operationalized through both physiological measures and self-assessments, predicted more negative attitudes toward immigrants (Aarøe et al., 2017). However, xenophobia is not a one-dimensional feature. For example, Faulkner et al. (2004) showed that Canadians had more negative attitudes toward foreigners from subjectively more distant and unknown countries (Mongolia and Peru) than toward subjectively closer and better known ones (Poland and Taiwan), and that participants under high disease-salience conditions expressed less positive attitudes toward foreign, but not familiar, immigrants and were more likely to endorse policies that would favor the immigration of familiar rather than foreign peoples.

Furthermore, it seems that one’s own health status mediates BIS (re)activity, i.e., that an organism’s physiological needs fine tune BIS activation. For example, recently and frequently ill people showed greater activation of the BIS (Stevenson et al., 2009; Miller and Maner, 2012; Murray et al., 2019), pregnant women expressed more ethnocentrism in the first trimester, presumably due to immunosuppression (Navarrete et al., 2007; however, the link between progesterone and disgust has been questioned; see Jones et al., 2018), and individuals who possess gene variants associated with greater susceptibility to certain infectious diseases and poorer immunological function reported lower levels of extraversion and openness to experience, as well as higher levels of harm avoidance (MacMurray et al., 2014; Napolioni et al., 2014). These variations in BIS reactivity imply that certain populations might provide valuable deeper insight into the inner workings of the BIS, and isolated populations are certainly among them.

### Croatian Island Isolates: The Rationale for This Study

When it comes to infectious disease propagation, living in an isolated area such as an island, is both a “blessing” and a “curse,” as it brings both the benefits and dangers of living in isolation. On the one hand, isolation can spare the whole community from being exposed to a pathogen, but on the other hand, this leads to a decreased population immunity, and when a deadly pathogen is re-introduced after a long time, there are no remaining immune individuals (Rudan, 2006). Isolated communities have, throughout history, been hit the hardest when some new infectious disease invades their village or island, leading to numerous deaths (Whittaker, 2018). Their lack of immunity caused by few contacts with the outside world, limited nutrition, household size, drinkable water, and access to sanitation, and healthcare availability among others, all contribute to rapid, and often deadly, spread of disease. It has been suggested that this might have contributed to entire civilizations being wiped out, for instance the inhabitants of the Easter Island and the cultures of Mayas and Aztecs in central America (Rothman and Greenland, 1998). Therefore, it can be speculated that the BIS would be more reactive among isolated populations.

With 48 populated islands, whose populations vary from 1 to 19,383 inhabitants, Croatia provides a fruitful testing ground for this hypothesis, as all other relevant variables which might influence the prophylactic behaviors and related cognitions and emotions, such as the dominant culture, religious beliefs, or the health care system, are equal among islanders and mainlanders. Croatian islands have been inhabited since the time of ancient Greece, and because of their location on important maritime routes, their inhabitants where exposed to various influences, from Rome and Byzantium, the Turkish and Austrian Empires, to the Venetian Republic and France. However, ships did not carry only goods: in addition to the benefits of trade, islanders also experienced numerous epidemics including plague, cholera, leprosy, and malaria and were faced with the need to avoid, prevent, or mitigate epidemics of infectious diseases (Cvetnić, 2014).

Numerous instances of plagues in the Adriatic area have been documented in historical archives, dating from as early as 160 AD. The “black death” ravaging European populations throughout middle ages found its way to Croatian islands by maritime routes (Bačić, 2007). Interestingly, the “black death” outbreak which took a huge toll on the European population in 1348 also brought some important epidemiological insights: it has been noted that outbreaks usually take place after the arrival of ships from distant locations, and thus a mandatory isolation of people and goods from those ships was proscribed. In fact, the world’s oldest known quarantine dates back to 1377 in Dubrovnik. The word “quarantine” stems from the word *quarranta*, meaning 40, which is the number of days that travelers arriving to Dubrovnik port had to spend in isolation. Aside from specialized quarantine units called *lazarettos*, some of the smaller Croatian islands in the middle ages were designated as locations for self-isolation and to this day, they bear names like *Gubavac*, meaning leper. There were instances in history when trading between the islands and the mainland was prohibited in order to prevent the spread of a disease. The outbreak in 1617 had the greatest impact on inhabitants of Korčula island: it was brought by sailors on a Venetian ship, spread fast among the local community, eventually eradicating whole lineages of families (Bačić, 2007).

Kralj-Brassard (2016) described the 17th century Dubrovnik Republic as being under constant threat from plague and other contagious diseases coming mostly from the neighboring regions of the Ottoman Empire. However, due to a well-organized system of public health measures against plague, developed and tested for centuries, such as the famous Dubrovnik’s *cordon sanitaire*, the number of outbreaks was smaller, their duration shorter, and the scope of contagion limited in comparison with the neighboring regions under Venetian or Ottoman rule. Along with isolating the sick and the travelers, islanders have also been known to self-isolate immediately upon hearing the news of new infections: an interesting example took place in the 19th century on the island of Hvar, where citizens who owned ships self-isolated on their vessels in the city harbor, and stayed there through the entire epidemic of cholera with only scarce food and water supplies, and yet all survived, while the ones who stayed in the city were decimated (Baras, 2020).

Another infectious disease threatening inhabitants of Adriatic islands was malaria. As early as 1420, the Korčula Statute forbade the people of Korčula to sail on the river Neretva, under the threat of punishment of losing all their property. This was a surprisingly appropriate public health measure against malaria—as it legally bound the residents of Korčula to avoid the mosquito-infested Neretva river estuary. Interestingly, Italian travel writer Alberto Fortis wrote in 1774 that he learned from a priest in the Neretva area that malaria is caused by mosquito bites—this was 125 years before Ronald Ross discovered that mosquitos transmit malaria (Eterović, 1994).

Later in history, Croatian islands witnessed surges of cholera, typhus, dysentery, scarlet fever—mostly brought by soldiers coming home from various regions of the Austro-Hungarian empire during WWI. Again, an elaborated set of measures was put in place: special gendarmerie squads patrolling the areas designated for isolation, disinfection protocols, social distance measures, school closures, even mass vaccination campaigns were organized when possible, exemplified by the mandatory vaccination against smallpox on the island of Krk (Kirinčić, 2019.

From the perspective of the BIS theoretical framework, these historical events also bring to light another interesting element, which can be found even in the epidemiological news section of the official *Journal of the Croatian Medical Association* (*Liečnički viestnik*, dating back to 1877) in which overtly xenophobic descriptions are used for certain groups perceived as disease carriers (e.g., “wandering gypsies,” “migrant folks,” “their folks” vs. “our folks,” “dirty ones,” etc.).

Given these specific geomorphological, economic, and demographic characteristics, as well as the historical heritage of Croatian islands, their inhabitants certainly represent an interesting study population, and have often been in the focus of geneticists and epidemiologists (see e.g., Rudan et al., 1999; Vitart et al., 2006). As for recent history, an extensive epidemiological study of several infectious diseases (salmonellosis, streptococcal angina, varicella, and scabies) on 10 Croatian islands (Krk, Cres, Lošinj, Rab, Pag, Brač, Hvar, Korčula, Vis, and Lastovo) between 1989 and 1998 showed that in comparison with the Croatian general population, epidemics on islands were less frequent, but of much greater intensity, especially in smaller and very isolated communities (Rudan et al., 2002). Even nowadays, while not isolated in the true meaning of the word, Croatian island towns are still relatively small, often with insufficient healthcare, far away from mainland hospitals, and with modest food supply if further from the coast, especially during off-season (Skračić, 2013).

With lockdown in place during the data collection for this study, islanders were isolated more than ever. Commuting restrictions were put forward, ferries to the mainland were scarce, and health services weren’t equipped for an outbreak, making existing disease threat even worse. Even though Croatian islands have long been known as tourist hotspots, and most Croatian islanders financially depend on tourism, during the lockdown of spring 2020, there were anecdotal reports of locals calling the police and reporting seeing “foreigners on the seafront.”

Thus, the aim of this study was to explore COVID-19 health anxiety and other BIS-related emotions and cognitions, such as germ aversion, perceived vulnerability to disease, and pathogen disgust in their relation to: (a) negative emotions toward strangers; (b) willingness to punish those who do not adhere to COVID-19 preventive measures; and (c) preferred interpersonal distances. Furthermore, we opted to test the prediction that living in an isolated area contributes significantly to such emotions and cognitions. Additionally, in order to test the possible effect of COVID-19 pandemic on BIS-related emotions and cognitions, we compared the current scores with the ones obtained in similar samples before the pandemic.

## Materials and Methods

### Participants

A total of 805 people, aged 16–71 (*M* = 35.52, SD = 11.96) participated in the study. Overall, there were 639 female and 166 male participants. Out of the 768 participants who indicated their place of living, there were 412 mainlanders and 356 islanders. Amongst mainlanders, aged 17–67 (*M* = 34.04, SD = 11.64), there were 326 female and 85 male participants, while amongst islanders, aged 16–71 (*M* = 37.53, SD = 11.96), there were 284 female, and 69 male participants.

On average, the islanders lived in larger households [*F* (1, 756) = 7.75; *p* < 0.001], including more young children [*F* (1, 722) = 22.45; *p* < 0.001], had lower monthly income [*F* (1, 756) = 88.91; *p* < 0.001], and as expected, their settlements had fewer inhabitants [*F* (1, 761) = 1063.94; *p* < 0.001]. Participants in our sample estimated their islands to have had an average of 5,900 inhabitants, and the settlement they currently lived in to have had an average of 1,700 inhabitants.

Importantly, islanders in our sample reported having fewer symptoms of respiratory infections in the period preceding the study [*F* (1, 767) = 28.83; *p* < 0.001], which is an indirect indicator of our assumption that they are indeed less exposed to a whole range of respiratory viruses than the mainlanders.

#### Pre-pandemic Samples

For the comparison of BIS-related variables before the pandemic with the ones collected for the purposes of this study, we used data from two of our earlier databases (collected online during 2017 and 2019; unpublished data). A total of 957 participants (351 men, 606 women) were comparable to the current sample in age (range 18–88; *M* = 31.56, SD = 12.56), and economic status (the SES variables were operationalized differently, so a direct statistical comparison is not possible, but the vast majority, approx. 60% of participants in both samples reported having an average income and about 30% reported having an above average income). However, for these samples, we do not have the information whether they lived on an island or on the mainland.

### Procedure

The link to the online questionnaire was shared on various social networks, and special attention was given to recruiting islanders through local Facebook groups. The data were gathered during April and May 2020, during the first wave of the COVID-19 pandemic. At that time, the Croatian government implemented a rather restrictive set of measures, restricting travel between counties, outside of special circumstances, social gatherings were restricted to a maximum of 10 people from a maximum of two households, schools went online, grocery shops worked reduced hours, and restaurants, pubs, and cafes were closed, as were all non-essential facilities. In fact, according to the Oxford COVID-19 government response tracker at that time, Croatia ranked as the strictest country on the scale and had the highest stringency index (Hale et al., 2020). Participants read the informed consent and if they agreed to participate, by clicking the “agree” button they were directed to the questionnaire. The first part of the questionnaire consisted of demographic information: gender, age, marital status, education, employment status (if unemployed, they were also asked if they had lost their job since the beginning of the lockdown), household size (including how many children under the age of 12), and household monthly income. They were then asked about their place of living, how many citizens it has, whether it can be considered a tourist hotspot, and if their place of residence is on the mainland or on an island.

If participants were islanders, they were then asked further questions: the size of the population of their island, how the island is connected to the mainland (ferry, catamaran, bridge etc.), how often the connections from island to mainland usually run, and how often they ran during lockdown.

If participants were mainlanders, they were asked whether they lived in a city or in a village. For villagers there were also additional questions: how far away the first bigger town the village is, how often public transport runs between their village and said town, and how often during the pandemic. Originally, we intended to test if there are any differences in BIS-related variables between residents of villages, where the density of population is low, and city dwellers. However, the sample size of people living in rural areas was too small to conduct a meaningful analysis.

The rest of the survey was the same for all participants. First, they were asked several questions concerning coronavirus—whether they, or any of their family members, are at an increased risk of contracting coronavirus and/or developing a complicated clinical presentation of COVID-19 disease, and whether they or anyone they know tested positive for coronavirus. They were also asked if and how has their daily life changed since the beginning of the pandemic. In addition, participants were asked to check the symptoms they have experienced during the last three weeks (if any). The symptoms listed were: stuffed nose, sneezing, sore throat, coughing, runny nose, headache, shivering, weakness/nausea. Afterward, they were asked to fill out various questionnaires to assess their COVID-19 anxiety, inclination toward punishing those who do not adhere to preventive COVID-19 measures, perceived vulnerability to disease, emotions toward strangers, conservatism, preferred interpersonal distances, and pathogen disgust proneness.

### Materials

#### COVID-19 Anxiety Scale

As a measure of COVID-19 anxiety, we used a COVID-19 concerns scale (Lauri Korajlija and Jokić-Begić, 2020). The scale consists of 5 items depicting various concerns regarding the impact of coronavirus on health including perceived likelihood of infection, perceived danger of COVID-19, and others. Participants had to indicate the extent to which an item relates to them on a scale ranging from 1 (not at all) to 5 (very much). The scale has good reliability (Cronbach α = 0.78).

#### Perceived Vulnerability to Disease

Perceived vulnerability to disease was measured using the scale developed by Duncan et al. (2009). This scale has 15 items that constitute two subscales: the Perceived infectability subscale (7 items) and Germ aversion subscale (8 items). Participants have to indicate their agreement with the items on a 1 (Strongly disagree) to 7 (Strongly agree) scale. The scale had very good overall reliability (Cronbach α = 0.82). The same was true for perceived vulnerability to disease subscale (α = 0.87), while germ aversion had good reliability (α = 0.74).

#### Disgust

Disgust proneness was measured using The Pathogen subscale of the Three Domains of Disgust Scale developed by Tybur et al. (2009). It has three subscales: pathogen disgust, moral disgust, and sexual disgust, but in this study only the pathogen disgust subscale was used. The subscale has 7 items describing situations that are considered disgusting, as they signal pathogen threat, and participants have to rate the items on a scale of 0 (not at all disgusting) to 6 (extremely disgusting) with three being a neutral value. The subscale had good reliability in this study (α = 0.74).

#### Conservatism

Social conservatism subscale of the 12-item Social and Economic Conservatism Scale (SECS) (Everett, 2013) was used to measure participants’ conservatism. The subscale consists of 7 items (abortion, army and national security, religion, traditional marriage, traditional values, family, and patriotism) and participants were asked how they feel about each item on a scale of 0–100, with 0 meaning “very negative”, 100 meaning “very positive” and 50 meaning “not negative nor positive”. The subscale yielded very good reliability, α = 0.801. The economic conservatism subscale was omitted, as it has previously been shown that this construct is not comparable between Croatian and United States samples (thus, the validity of this subscale for use in Croatian samples is debatable; Mrakovčić and Buršić, 2017).

#### Preferred Interpersonal Distances

To measure preferred interpersonal distances, we used a graphic task developed by Sorokowska et al. (2017). This instrument measures three different preferred interpersonal distances—preferred distance to a stranger (social distance), an acquaintance (personal distance), and a close person (intimate distance). Participants are presented with two human figures, one on each end of the scale, labeled A and B. They were instructed to imagine that they were person A and to rate how close the person B could approach them, in order for them to still feel comfortable. The distance on the scale ranged from 0 to 220 cm, and each participant gave three assessments: if the person B was a stranger, an acquaintance, or a close person. Before the COVID-19 pandemic, on a sample size of 614 participants, the values obtained in Croatia for preferred interpersonal distances were: *M* = 108.86 (SD = 28.74) for strangers, *M* = 89.61 (SD = 24.06) for acquaintances, and *M* = 76.16 (SD = 23.84) for close persons (Sorokowska et al., 2017).

#### Negative Intergroup Emotions Scale

This scale was developed by Stephan et al. (1999) and is used as a measure of affective component of attitude. It consists of six positive and six negative emotions and participants have to indicate the extent to which they feel a certain emotion toward strangers on a 1 (not at all) to 7 (very much) scale. Before calculating the final score, positive emotions need to be recoded so that the higher result indicates more negative emotions. In this study, the scale had a good reliability of α = 0.77.

#### Inclination to Punish Non-adherence to COVID-19 Preventive Measures

Participants were presented with two statements about their inclination toward punishing those not abiding by the rules set by the government (“I want the government to harshly punish everyone who is breaking the rules and is not staying at home” and “It is essential for the government to punish people who don’t respect the rules of social distancing”) and they had to indicate their agreement with the statements on a scale from 1 (“Strongly disagree”) to 5 (“Strongly agree”).

## Results

The data was analyzed using SPSS Statistics for Windows, Version 26.0 (IBM Corp, 2019 Released 2019) and JAMOVI (The jamovi project, 2021).

### Predictors of Negative Emotions Toward Foreigners

A two-stage hierarchical multiple regression analysis was conducted with demographic variables (including the geographical location: island vs. mainland) entered at stage one. Here we included conservatism, to control for the fact that islanders tend to be more conservative than mainlanders [Državno izborno povjerenstvo republike Hrvatske (DIP), 2020]. The BIS-related variables entered at stage two included: the perceived infectability and the germ aversion subscales of the Perceived vulnerability to disease scale, the pathogen disgust subscale of the Three domain disgust scale.

As can be seen from Table 1, the hierarchical multiple regression revealed that variables entered at stage one contributed significantly to the regression model, [*F* (5, 512) = 5.84, *p* < 0.001] and accounted for 5.4% of the variation in negative emotions toward foreigners. The only single significant variable contributing to this was the geographical location (islands vs. mainland). Introducing the BIS variables explained an additional 4.5% of variation in the dependent variable and this change in *R*^2^ was significant, *F* (8, 509) = 6.98, *p* < 0.001. When all eight independent variables were included in stage two of the regression model, the most important predictor of negative emotions toward foreigners was pathogen disgust, followed by perceived infectability, geographical location, age, and gender.

**TABLE 1:** Summary of hierarchical regression analysis for variables predicting negative emotions toward foreigners.

**Variable**	**B**	***t***	***sr***	***R***	***R*^2^**	**Δ*R*^2^**
**Step 1**						
Gender	–0.080	–1.867	–0.080	0.232	0.054	0.054
Age	–0.078	–1.808	–0.077			
Settlement population size	–0.039	–0.630	–0.027			
Geographical location	0.181	2.683**	0.115			
Conservatism	0.020	0.46	0.200			
**Step 2**						
Gender	–0.107	−2.150*	–0.091	0.314	0.099	0.045
Age	–0.088	−2.171*	–0.092			
Settlement population size	–0.053	–0.931	–0.039			
Geographical location	0.168	2.544*	0.107			
Conservatism	0.002	0.041	0.002			
Germ aversion	0.021	0.418	0.018			
Perceived infectability	0.122	2.82*	0.119			
Pathogen disgust	0.167	3.49***	0.147			

***p < 0.001. **p < 0.01. *p < 0.05.

### Predictors of Inclination to Punish Those Who Do Not Adhere to COVID-19 Preventive Measures

A two-stage hierarchical multiple regression analysis was conducted with demographic variables entered at stage one. Here we also included conservatism, to control for the fact that islanders tend to be more conservative than mainlanders. The BIS-related variables entered at stage two included: the perceived infectability and the germ aversion subscales of the Perceived vulnerability to disease scale, the pathogen disgust subscale of the Three domain disgust scale, and the COVID-19 anxiety scale.

As can be seen from Table 2, the hierarchical multiple regression revealed that variables entered at stage one contributed significantly to the regression model, [*F* (5, 512) = 14.89, *p* < 0.001] and accounted for about 10% of the variation in willingness to punish rule-breakers. Conservatism had a significant impact here, explaining 6.4% of variance in the dependent variable. Introducing the BIS variables explained an additional 18% of variation in the dependent variable and this change in *R*^2^ was significant, *F* (9, 508) = 21.71, *p* < 0.001. When all eight independent variables were included in stage two of the regression model, the most important predictor of willingness to punish those who do not adhere to the preventive measures was COVID-19 anxiety which uniquely explained 8.2% of the variation in the dependent variable, followed by conservatism and gender.

**TABLE 2:** Summary of hierarchical regression analysis for variables predicting inclination to punish those who do not adhere to COVID-19 preventive measures.

**Variable**	**β**	***t***	***sr***	***R***	***R*^2^**	**Δ*R*^2^**
**Step 1**						
Gender	0.162	3.86***	0.162	0.314	0.099	0.099
Age	0.007	0.17	0.007			
Settlement population size	–0.062	–0.92	–0.039			
Geographical location	–0.076	–1.14	–0.048			
Conservatism	0.260	6.02***	0.252			
**Step 2**						
Gender	0.105	2.76**	0.104	0.527	0.278	0.179
Age	–0.069	–1.78	–0.067			
Settlement population size	–0.096	–1.59	–0.060			
Geographical location	–0.070	–1.17	–0.044			
Conservatism	0.182	4.54***	0.171			
Germ aversion	0.183	4.08***	0.154			
Perceived infectability	0.019	0.48	0.018			
Pathogen disgust	0.063	1.47	0.055			
COVID-19 anxiety	0.319	7.59***	0.286			

***p < 0.001. **p < 0.01.

### Predictors of Preferred Interpersonal Distances

Three two-stage hierarchical multiple regression analyses were conducted with demographic variables (including the geographical location: island vs. mainland) entered at stage one, and the BIS-related variables (the perceived infectability and the germ aversion subscales of the Perceived vulnerability to disease scale, the pathogen disgust subscale of the Three domain disgust scale, and COVID-19 anxiety scale) were entered at stage two. The dependent variables were preferred interpersonal distances: social distance (stranger), personal distance (acquaintance), and intimate distance (close person). The results can be seen in Table 3.

**TABLE 3:** Summary of hierarchical regression analyses for variables predicting preferred interpersonal differences.

**Dependent variable**	**Social distance (stranger)**					**Personal distance (acquaintance)**					**Intimate distance (close person)**				
**Predictors:**	**β**	***t***	***sr***	***R*^2^**	**Δ*R*^2^**	**β**	***t***	***sr***	***R*^2^**	**Δ*R*^2^**	**β**	***t***	***sr***	***R*^2^**	**Δ*R*^2^**
**Step 1**															
Gender	0.030	0.68	0.030	0.032	0.032	0.071	1.64	0.071	0.032	0.032	0.112	2.49*	0.112	0.019	0.019
Age	0.105	2.36**	0.103			0.167	3.77***	0.164			0.045	0.99	0.044		
Settlement population size	0.067	0.96	0.042			0.016	0.23	0.010			–0.054	–0.77	–0.034		
Household size	0.103	2.29**	0.100			0.021	0.46	0.020			–0.016	–0.34	–0.015		
Geographical location	0.129	1.88*	0.082			–0.009	–0.13	–0.006			–0.004	–0.05	–0.002		
**Step 2**															
Gender	–0.009	–0.22	–0.009	0.152	0.120	0.032	0.77	0.032	0.152	0.12	0.080	1.82	0.079	0.085	0.067
Age	0.049	1.15	0.047			0.105	2.48*	0.102			–0.002	–0.035	–0.002		
Settlement population size	0.047	0.72	0.029			–0.004	–0.06	–0.002			–0.065	–0.95	–0.041		
Household size	0.094	2.21*	0.090			0.015	0.35	0.014			–0.021	–0.47	–0.021		
Geographical location	0.128	1.97*	0.081			–0.007	–0.12	–0.005			–0.008	–0.11	–0.005		
Germ aversion	0.222	4.85***	0.198			0.193	4.20***	0.172			0.121	2.52*	0.110		
Perceived infectability	0.048	1.11	0.045			0.022	0.51	0.021			0.022	0.47	0.021		
Pathogen disgust	0.029	0.65	0.027			0.018	0.39	0.016			0.067	1.42	0.062		
COVID-19 anxiety scale	0.186	4.09***	0.167			0.231	5.08***	0.208			0.171	3.57**	0.155		

***p < 0.001. **p < 0.01. *p < 0.05.

#### Social Distance (Stranger)

As can be seen from Table 3 (first column), the hierarchical multiple regression revealed that variables entered at stage one contributed significantly to the regression model [*F* (5, 511) = 3.38, *p* < 0.01] and accounted for 3.2% of the variation in preferred social distance. Single variables with significant contribution at this step were age, household size, and geographical location. Introducing the BIS variables explained an additional 12% of variation in preferred social distance and this change in *R*^2^ was significant, *F* (9, 507) = 10.09, *p* < 0.001. When all nine independent variables were included in stage two of the regression model, the most important predictors of preferred social distance were germ aversion and COVID-19 anxiety scale, followed by household size and geographical location.

#### Personal Distance (Acquaintance)

As can be seen from Table 3 (second column) the hierarchical multiple regression revealed that variables entered at stage one contributed significantly to the regression model, *F* (5, 511) = 3.41, *p* < 0.01, and accounted for 3.2% of the variation in preferred personal distance. Age was the only variable with significant contribution at this step. Introducing the BIS variables explained an additional 12% of variation in preferred social distance and this change in *R*^2^ was significant, *F* (9, 507) = 10.09, *p* < 0.001. When all nine independent variables were included in stage two of the regression model, the most important predictors of preferred social distance were COVID-19 anxiety (accounting for 4.3% of total variance) and germ aversion (accounting for almost 3% of total variance), followed by age.

#### Intimate Distance (Close Person)

As can be seen from Table 3 (third column), the hierarchical multiple regression revealed that variables entered at stage one did not contribute significantly to the regression model, *F* (5, 486) = 1.85, *p* = 0.10. Introducing the BIS variables explained an additional 7% of variation in preferred intimate distance which yielded a significant change in *R*^2^, *F* (9, 482) = 5.01, *p* < 0.001. When all nine independent variables were included in stage two of the regression model, the only important predictors of preferred intimate distance were COVID-19 anxiety and germ aversion. It is interesting to note though that germ aversion and COVID-19 anxiety were the only two predictors with significant contributions to the preference for all three interpersonal distances.

To check for possibility that geographical location serves as a moderator between the BIS-related variables and criterion variables, we re-ran all the analyses (using *medmod* module in JAMOVI), adding interactions between BIS-variables and geographical location (island vs. mainland). Out of 15 possible interactions (three BIS-related variables combined with five criterions: negative emotions toward foreigners, willingness to punish non-adherence, and three types of interpersonal differences), only two proved significant: geographical location moderated only the relationship between germ aversion and negative emotions toward strangers (*b* = 0.234, *p* = 0.027) and between pathogen disgust and negative emotions toward strangers (*b* = 7.56, *p* = 0.025) with those associations in both cases being more pronounced among islanders than mainlanders.

**FIGURE 1:**
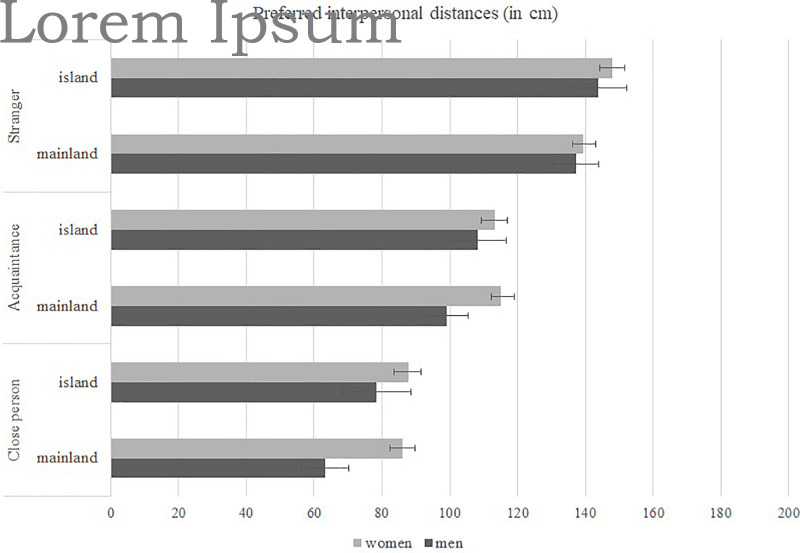
Preferred interpersonal distances (in cm) as a function of geographical location (island vs. mainland) and gender.

Since preferred interpersonal distances have previously been shown to depend not only upon culture, but gender and context as well (see Vranić, 2003; Iachini et al., 2016; Sorokowska et al., 2017), we opted to explore them in more detail. In order to do so, we conducted a repeated measures MANOVA with gender (men/women) and geographical location (mainland/island) as between-subjects source of variance and type of interpersonal distance (social/personal/intimate distance) as a within-subject source of variance. There was a significant main effect of gender, with women overall preferring larger interpersonal distances [*F* (1, 643) = 5.51; *p* < 0.02]. With regard to geographical location (mainland/island), there was no significant main effect on preferred interpersonal distances [*F* (1, 647) = 2.11; *p* = 0.14]. However, there was a significant interaction between geographical location and type of interpersonal distance [*F* (1, 647) = 3.12; *p* = 0.04], stemming from the fact that islanders preferred larger social distances than mainlanders, but there were no differences in preferred personal and intimate distances. Furthermore, as expected, there was a significant within-subjects effect, with the preferred social distances being the largest, followed by personal distances and intimate distances were the smallest [*F* (1, 647) = 415.37; *p* < 0.001]. This can be seen in Figure 1.

### Is There a Difference in BIS-Related Variables From Before COVID-19?

To answer this question, we compared these scores with the ones obtained before the COVID-19 pandemic. The data for interpersonal distances were collected as a part of large cross-cultural study (see Sorokowska et al., 2017) and the data for germ aversion, perceived infectability, and pathogen disgust scales were collected online during 2017 and 2019 (Hromatko, unpublished data). As can be seen from Tables 4, 5, scores on relevant variables (germ aversion, perceived infectability, pathogen disgust, and preferred interpersonal differences) were significantly higher in this sample, as compared to our pre-pandemic samples. As expected, women expressed significantly higher levels of pathogen disgust, germ aversion, and perceived infectability than men, and there was only one significant gender × time interaction: only men showed an increase in perceived infectability during the pandemic. Additionally, to control for the possibility that the shifts from pre-pandemic to pandemic scores were driven by the larger proportion of women in our samples, we conducted separate analyses for men and women. The effect remained robust for men, as they had significantly higher pandemic than pre-pandemic scores in pathogen disgust (*F* = 4.06, *p* = 0.04), germ aversion (*F* = 35.48, *p* < 0.001), and infectability (*F* = 7.51, *p* = 0.01). Women had significantly higher pandemic than pre-pandemic scores in pathogen disgust (*F* = 10.19, *p* < 0.001), and germ aversion (*F* = 113.87, *p* < 0.01), with no change in perceived infectability (*F* = 0.02, *p* = 0.88). Considering that we did not have information about the geographical location (island vs. mainland) of the participants in our pre-pandemic samples, but it is reasonable to assume that those were mostly mainlanders, we re-ran the same analyses without islanders in the pandemic sample, and the results remained the same. This was expected, as there were no overall differences in BIS-related variables between mainlanders and islanders (*F*_pathogen.disgust_ = 0.06, *p* = 0.80; *F*_germ.aversion_ = 1.75, *p* = 0.19; *F*_infectability_ = 2.14, *p* = 0.14).

**TABLE 4:** BIS-related measures before/during the COVID-19 pandemic.

	**Pathogen disgust**		**Germ aversion**		**Infectability**	
	**Men**	**Women**	**Men**	**Women**	**Men**	**Women**
	***M* (SD)**	***M* (SD)**	***M* (SD)**	***M* (SD)**	***M* (SD)**	***M* (SD)**
Before pandemic	3.438 (1.185)	3.966 (0.992)	3.577 (1.077)	3.800 (1.027)	2.782 (1.115)	3.189 (1.342)
During pandemic	3.763 (2.201)	4.109 (1.086)	4.216 (1.028)	4.459 (1.073)	3.079 (0.976)	3.199 (1.098)

**ANOVA**	***F* (1, 1597)**	**Partial η*^2^***	***F* (1, 1640)**	**Partial η*^2^***	***F* (1, 1642)**	**Partial η*^2^***

Time (pre-pandemic/pandemic)	10.616***	0.007	111.26***	0.064	4.939*	0.003
Gender (men/women)	36.779***	0.023	14.301***	0.009	14.483***	0.009
Time × gender	1.591	0.001	0.025	0.000	4.286*	0.003

***p < 0.001. **p < 0.01. *p < 0.05.

**TABLE 5:** Preferred physical distances before/during the COVID-19 pandemic.

	**Before the pandemic (2016; *N* = 614)**		**During the pandemic (2020; *N* = 684)**		
	***M***	**SD**	***M***	**SD**	***t***
Stranger (social distance)	108.86	28.74	142.10	55.7	13.29***
Acquaintance (personal distance)	89.61	24.06	112.17	55.24	9.32***
Close person (intimate distance)	76.16	23.84	83.45	62.97	2.69**

***p < 0.001; **p < 0.01.

## Discussion

The main aim of this study was to determine whether certain social cognitions, namely negative emotions toward strangers, inclination to punish rule breakers (those who do not adhere to COVID-19 preventive measures), and preferred interpersonal distances can at least partly be explained as a result of BIS activation. Furthermore, we opted to gather some insights into the workings of this system in semi-isolated populations of islanders. As we have already stated, we cannot claim that the population of Croatian islanders nowadays is isolated in a classical anthropological sense. However, they do retain some aspects of a more traditional lifestyle, with larger family units within smaller communities and limited connections with the mainland, depending on the island, the frequency of marine lines varies between several lines daily to several weekly, which does make them a semi-isolated population. Furthermore, Croatian island populations were recognized as one of the best-characterized isolate resources in Europe, and as such are included in the “European Special Population Research Network,” a project funded by the European Commission and aimed at studying the determinants of human health and disease (Rudan, 2006).

There are also advantages to the fact that the islanders in our sample share a lot in common with the country’s mainlanders, as this makes the two groups more comparable, and we can make more specific claims regarding our findings. Local folklore and variations in norms and customs notwithstanding, these participants do share a common culture with their mainland compatriots in the form of sharing the same language, religious views, and political system among others, meaning that alternative explanations of our findings are less likely. For example, we can exclude different religious practices as an intervening variable mediating the relation between perceived vulnerability to disease or pathogen disgust and preferred social distance.

Islanders in our sample reported having significantly fewer symptoms of respiratory illnesses in the period preceding the study, which we believe is an important indicator of their reduced exposure to various “common-cold” causing viruses. This was important for our hypothesis, because, as we have elaborated earlier, isolated populations are shielded from exposure to various pathogens, but that also makes them more vulnerable once a new pathogen finds its way to the population. Pathogens are more likely to spread faster, partly because of this lack of previous exposure and subsequent lack of population immunity, and partly because of the way of life. Islanders live in larger, multigenerational households and they are more likely to gather at the same “hotspots” in their local communities, seeing as there is often only one post-office, one grocery shop or market, one church, one general practitioner, or family practice, covering medical needs of all generations, which facilitates the spread of a contagious disease. Thus, the finding that islanders experienced less respiratory symptoms in the period preceding the COVID-19 pandemic than the mainlanders does not imply they have greater biological predisposition to fight off infection; rather it indicates that they were indeed shielded from a variety of other, common respiratory viruses. City dwellers are more exposed to them, as they cannot avoid crowded, closed spaces, such as public transportation or large office buildings with inappropriate ventilation systems. Recent studies have suggested that earlier exposure to other human coronaviruses (often called common cold coronaviruses) makes one if not immune, then at least less likely to develop a complicated clinical presentation of COVID-19 (Guthmiller and Wilson, 2020; Mateus et al., 2020; Sagar et al., 2021). Another non-biological reason why islanders might be at greater risk if they contract the infection is that it will take them longer to get medical help, since they need to reach the mainland, either by marine routes or, in cases of extreme emergency, by air. This was the basis for our prediction that islanders should show greater BIS reactivity.

### Negative Emotions Toward Foreigners

As stated earlier, xenophobia might be at least partially explained as a consequence of disease avoidance, and similar patterns of xenophobic escalations have been reported in previous epidemics, as well as this one (Sorokowski et al., 2020). Our analyses (see Table 1) showed that, after controlling for conservatism, the only significant variable contributing to negative emotions toward foreigners in the first step of the analysis was place of residence: living on an island correlated significantly with higher animosity toward strangers. This predictor remained significant even after the introduction of a set of variables pertaining to the BIS, when the most important single predictor of negative emotions toward foreigners became pathogen disgust, followed by perceived infectability. As expected, participants with higher scores on BIS variables expressed more negative emotions toward foreigners. Women and older participants expressed fewer negative emotions toward foreigners.

As far as avoidance mechanisms are concerned, these findings are in line with the notion that, especially during the pandemic, when cues of risk of infection are abundant, xenophobic attitudes might serve as a steering wheel, keeping one from coming into close contact with a possible disease carrier. Our prediction that this shall be more pronounced among islanders, was confirmed. As we have argued in the introduction, we do not believe that this difference would be easily attributed to islanders’ lack of experience with foreigners, as most of them in one way or another depend upon tourism, and during the summer Croatian islands become tourist hotspots. Even though contemporary humans live in a global world, exposed to various cultures and as some have argued (e.g., Ackerman et al., 2020) would have no use of applying the same small-scale society heuristics of foreigners being the potential carriers of new pathogen, we have witnessed that in the context of a global health crisis such as this one, it does not take much for a (re)activation of a sort of “mental *cordon sanitaire*.” The fact that scores on disgust sensitivity, germ aversion, and perceived infectability have risen significantly in comparison to pre-pandemic scores (Miłkowska et al., 2021; Stevenson et al., 2021) is in line with this notion. The distinction between the proactive and reactive aspects of outputs generated by the BIS (Ackerman et al., 2018) might be of special importance here, as there is no doubt that in this particular scenario, we are dealing with reactive responses. These responses are induced by the presence of information connoting an immediate infection risk, and even if the pathogen threat cannot be detected through our own sensory routes, due to a lack of obvious signs of infection, the awareness of the risk is still heightened.

As our study was conducted during a lockdown, accompanied by a rather strict set of measures, islanders were shielded within their small communities and likely felt threatened by the possibility of outsiders carrying the disease into their, at that time, rather closed communities. Mainlanders, especially those living in larger cities, still used public transport and continued interacting with strangers on a daily basis, often in crowded spaces, and thus may have been desensitized to such situations. Triggers for the BIS activation would have remained the same, but the intensity of their responses could have been attenuated, due to repeated exposure to situations in which contagion is possible. To illustrate this dynamic in a more anecdotal way, in March 2020 the capital of Croatia, Zagreb, was hit by the strongest earthquake since 1880, followed by numerous aftershocks (see e.g., Markušić et al., 2020), leaving over 1,900 buildings uninhabitable by the earthquake damage. Numerous citizens of the capital then tried to find refuge at the coastal parts of Croatia, which led to a public outcry by the locals worried that this will enable the propagation of COVID-19 contagion. Police had to intervene and prevent people from leaving the capital (HINA, 2020; Raić Knežević, 2020).

### Inclination to Punish Those Who Do Not Adhere to COVID-19 Preventive Measures

Humans have an evolved tendency to punish social-norm breakers (Krebs, 2008). For an interesting discussion about non-compliance with safety measures as a free-riding strategy and psychological mechanisms aimed at curbing free riding in context of COVID-19 pandemic, see Yong and Choy (2021). We predicted this tendency to punish non-adherence to preventive guidelines would be even more pronounced in situations such as this, where breaking certain rules designed to prevent the spread of a disease directly puts other members of the group in danger. Our results (Table 2) showed that conservatism and gender had a significant impact here, explaining 10% of variance in the dependent variable: conservatives and women were more likely to agree with the statements that government should punish severely those who do not adhere to COVID-19 preventive measures. Introducing the BIS variables explained additional 18% of variation in the dependent variable, and the most important predictor of willingness to punish those who do not adhere to the preventive measures was COVID-19 anxiety which uniquely explained 8.2% of the variation in the dependent variable, followed by germ aversion, conservatism, and gender. Expectedly, BIS variables were positively correlated with the dependent variable.

It should be noted that, unlike in some United States samples (see e.g., Calvillo et al., 2020; Corpuz et al., 2020), in our sample social conservatism correlated positively with the inclination to punish those who do not adhere to COVID-19 preventative measures. We expected a positive association, as the social component of conservatism aligns with traditionalism or the endorsement of traditional values, such as family, patriotism, loyalty, and norm-following. Also, the finding that conservatism differentially predicts COVID-19 preventative measures adherence in United States and Croatian samples is in line with the finding that this particular scale (Everett, 2013) has a different factorial structure in Croatian and United States samples (Mrakovčić and Buršić, 2017). As Calvillo et al. (2020) have suggested, the relationship between political ideology and threat perceptions may depend on issue framing by political leadership and media. Furthermore, when it comes to endorsement of authoritarian policies intended to mitigate the effects of the COVID-19, authoritarianism might be a more suitable psychological correlate than conservatism (Manson, 2020).

As for women, their higher inclination for punishment of those who do not adhere to COVID-19 preventive measures might be a result of their traditional role of homemakers: women are more likely to tend to children, sick, and elderly, and in this case, they are the ones who risk more by contracting the virus and potentially transmitting it to their families. For an in-depth review of these sex differences and their impact on pandemic leadership, pertaining to the fact that female leaders seem to be more focused on minimizing direct human suffering caused by the SARS-CoV-2 virus, while male leaders implement riskier short-term decisions, possibly aiming to minimize economic disruptions, see Luoto and Varella (2021). Furthermore, when making moral judgments, women are more likely to take into account the consequences of one’s actions, alongside the moral norms (Rothbart et al., 1986). Women on average, tend to be more anxious, and this was the case in our sample as well, with women having higher scores in COVID-19 anxiety. It has been shown earlier that people prefer less risk when the threat of illness was high (Prokosch et al., 2019). All these could have contributed to women’s higher inclination to punish those who do not behave responsibly, and those whose actions they might have perceived as being potentially harmful for their families.

Regarding this dependent variable, we didn’t find any differences between islanders and mainlanders. However, we are not convinced that this implies that there are no differences in BIS reactivity between these two populations. It is more likely that the measure itself was not elaborated enough to catch the subtle, non-formal ways of detecting and punishing social rule breakers. This was an *ad hoc* two-item measure, and both items pertained to the government’s role in implementing the punishment. In fact, a more nuanced scale, assessing one’s willingness to engage in non-formal ways of governing the behavior of others and streaming them into predefined modes of expected and acceptable behaviors would be a better choice, especially in the context of previous research showing that various dimensions of conservatism have different relations to parasite stress (Tybur et al., 2016). However, we were motivated to keep our questionnaire as short as possible in order to keep participants motivated.

### Preferred Interpersonal Distances

Since many diseases can spread by a simple touch (Schweon et al., 2013), and with our recent experience with a highly virulent aerosol-borne pathogen, it should not come as a surprise that increasing interpersonal distance has historically been an important aspect of behavioral adaptation against epidemics (Fenichel, 2013), and that similar adaptations have been observed among other species (Goodall, 1986; Schaller, 2011). It has even been suggested that cross-cultural differences in preferred interpersonal distances can be explained by parasite stress (see e.g., Sorokowska et al., 2017).

This was the rationale for choosing our final set of criteria: social, personal, and intimate distances. Here, we expected the largest effects of the BIS activation to be seen in preferred social distance, meaning the distance between oneself and a stranger. We expected smaller effects to be seen for preferred personal distance, meaning the distance between oneself and an acquaintance, and the smallest effects for the preferred intimate distance, meaning the distance between oneself and someone who is close to us.

As can be seen in Table 3, our predictors explained up to 15.2% of the variance in preferred interpersonal distances, leaving quite a large proportion of variance unexplained. However, it should also be noted that the variance in these dependent variables was greatly reduced due to the fact that there is an epidemic and that we have been instructed over and over again to keep a distance of minimum 2m. For example, for a stranger, 13.5% of our participants put the slider at the very end of scale (220 cm), which clearly indicates that the variability of true responses was artificially reduced with this scale. However, we wanted to be able to compare the results to the ones obtained before the pandemic, so we kept the original graphic scale (Sorokowska et al., 2017). Even with this limitation, we found a significant contribution of geographical location, with islanders preferring larger social distances. Household size and age were also significant, both positive, meaning that older people and those living in larger households preferred larger distances. This makes sense considering that in the case of COVID-19 age is one of the major risk factors for developing a severe form of illness. Furthermore, people living in larger households are more likely to have an older member of the family, or an immunocompromised one, or a small child in their care living with them, and are more motivated to reduce the risk of contagion. Furthermore, even though islanders in our sample lived in larger households, the contribution of living on an island was still a significant independent predictor of preference for larger social distance. Introducing the BIS variables explained an additional 12% of variation in preferred social distance. When all nine independent variables were included in stage two of the regression model, the most important predictors of preferred social distance were COVID-19 anxiety scale, followed by germ aversion, household size, and finally geographical location.

As for personal distances, the regression model revealed that in the first step age was the only variable with significant contribution. Again, older people preferred larger personal distances. The BIS variables explained an additional 12% of variance, with the most important predictors being COVID-19 anxiety (accounting for 4.3% of total variance) and germ aversion (accounting for almost 3% of total variance), followed by age. All these findings are in line with the idea that perceived risk of contracting a disease, along with parameters, such as age, which objectively put one in a riskier position, shall be associated with preference for keeping a safe distance. There was no significant contribution of the geographical location, which is not surprising, given the fact that on most islands almost everyone is at least an acquaintance, and their whereabouts during the lockdown were well-known which means that they do not pose such a contagion threat as potentially disease-carrying strangers. The same holds true for intimate distance. Here, the whole set of demographic variables failed to explain a significant proportion of the variance in the dependent variable, although gender as a single predictor was significantly correlated with women preferring larger intimate distances. Introducing the BIS variables explained an additional 7% of variance and when all nine independent variables were included in stage two of the regression model, the only important predictors of preferred intimate distance were COVID-19 anxiety and germ aversion. It is interesting to note that germ aversion and COVID-19 anxiety were the only two predictors with significant contributions to the preference for all three interpersonal distances, indicating that, even in interactions with familiar and close persons, the reactivity of the BIS still moderates the preferred physical distance.

Since there were some conflicting results in earlier literature regarding gender differences in preferred interpersonal distances (see e.g., Sorokowska et al., 2017 in comparison with Ozdemir, 2008 or Vranić, 2003), we opted to explore gender differences and their possible interaction with geographical location in greater detail. To that aim we conducted a repeated measures MANOVA with gender (men/women) and geographical location (mainland/island) as a between-subjects source of variance and type of interpersonal distance (social/personal/intimate distance) as a within-subject source of variance. We found a significant main effect of gender, with women preferring larger interpersonal distances across types of distances (see Figure 1). Sensitivity of peripersonal spaces to social aspects such as gender and age of the person approaching the participant, has also been stressed by Iachini et al. (2016). However, in our study, neither the gender nor age of the person approaching was specified and thus direct comparison of results is not possible. Our results are in line with the findings of Sorokowska et al. (2017) and we find their interpretation that women are more sensitive to social situations and tend to avoid dominant “invasions” of their personal space most likely.

With regard to geographical location (mainland/island), there was no significant main effect on preferred interpersonal distances. However, we found a significant interaction between geographical location and preference depending on the type of interpersonal distance: islanders preferred larger social distances than mainlanders, but there were no differences in preferred personal and intimate distances. This means that geographical location did not play a role in preferred distances from familiar and close persons—which we have already elaborated on earlier in the text. Furthermore, it should be noted that, along with other previously established variables (culture, social context, etc.) influencing preferred interpersonal distances, during a pandemic, there is a new element which should be taken into account. A recent study showed that wearing a mask reduces the subjective need for social distancing (Cartaud et al., 2020). In our particular task, however, this probably did not affect the results, as in spring 2020 mask wearing was practically non-existent in Croatia, with the exception of closed, public spaces, such as grocery shops, banks etc., mask wearing was neither mandatory nor recommended at the time.

### How Did the COVID-19 Pandemic Impact our Behavioral Immune Systems?

To answer this, we compared the scores in BIS-related variables obtained in this sample with some of our earlier pre-pandemic samples. Not surprisingly, scores on all relevant variables (germ aversion, perceived infectability, pathogen disgust, and preferred interpersonal differences) were significantly higher in this sample (see Tables 4, 5). These findings are in line with recent findings by Miłkowska et al. (2021) who found elevated levels of disgust sensitivity among women in a pandemic sample as compared to a pre-pandemic sample, and Stevenson et al. (2021), who found similar shifts in pre-pandemic to pandemic scores in core disgust and only marginally in germ aversion among several student cohorts. Our results differ from theirs regarding perceived infectability, which is probably a consequence of sample structure: their samples comprised of much younger participants than ours, which might have influenced participant vulnerability perception during the pandemic, seeing that older people are objectively more vulnerable to the SARS-CoV-2 infection. Furthermore, it seems that the shift toward higher infectability in our sample was driven by male scores, as there was a significant gender × time interaction with men perceiving themselves to be more vulnerable than before the COVID-19 pandemic, and women did not. Considering that men are more vulnerable to SARS-CoV-2 infection (Krams et al., 2020, 2021), and more likely to develop a complicated clinical presentation this finding seems to yet again reflect an adaptive shift in threat perception among men. This is especially relevant considering that a similar pattern has been observed in SARS and Middle East respiratory syndrome infections (Falahi and Kenarkoohi, 2021). As for the other two BIS-related variables, we found no interaction, only main effects of gender and time: in both pre-pandemic and pandemic samples, women showed higher levels of pathogen disgust and germ aversion than men, which is also in accordance with earlier research on sex differences in disgust sensitivity (see Al-Shawaf et al., 2018) and both men’s and women’s scores shifted significantly as a function of the pandemic.

Many of the BIS-related variables are usually operationalized as relatively stable traits, but these findings further underscore the notion that ecological and contextual elements modulate the expression of BIS components. We did not change the regular instructions on any of these scales nor did we instruct our participants to answer how they feel regarding their germ aversion and possible contamination now, as compared to how they usually feel, and yet the average scores shifted significantly compared to pre-pandemic scores. However, it should also be noted that all of our samples included disproportionately more women than men, and since there is an abundance of earlier work showing that women have greater disgust sensitivity (Al-Shawaf et al., 2018), we re-ran the pre-pandemic/pandemic analyses separately for men and women, and showed that shifts toward higher scores in all three BIS-related variables remained stable for men. However, this greater proportion of women in our pandemic sample might have skewed the results of regression analyses. Future research should take this into consideration, since from our experience, online studies without incentives for participants usually result in greater proportion of women. Another possible limitation of such comparison is measurement invariance. Our preliminary analyses suggest that the factor structures of the BIS-related questionnaires are stable in time, but we plan to explore this problem in a larger data set in ongoing research.

## Conclusion

In conclusion, we found that BIS variables contributed significantly to all dependent variables, including preferred interpersonal distances, social cognitions, and emotions. Those whose BISs were more reactive or those who felt higher levels of pathogen disgust, germ aversion, and perceived themselves as more likely to get infected, felt more negative emotions toward strangers, preferred to keep larger physical distances from them, as well as from acquaintances and close persons, and were more inclined to punish those who did not adhere to the social and official rules implemented in order to prevent the spread of COVID-19. Members of (semi)isolated populations, in this case islanders, likely express such avoidant tendencies more intensely, as they are more susceptible to infectious diseases, being less exposed to various viral vectors due to their lifestyle. Finally, even though our sample size does not allow us to draw any conclusions at the population level, average scores on all BIS measures have shifted toward significantly higher average scores, indicating the effects of globally heightened awareness of potential contamination cues in our environments and a sort of sensitization to pathogen threat. Observed together, these findings further corroborate the notion that the BIS is a highly contextually sensitive pathogen detection and avoidance system, at least partially underlying various social cognitions and patterns of interpersonal approach/avoidance motivations.

## Data Availability Statement

The raw data supporting the conclusions of this article will be made available by the authors, without undue reservation.

## Ethics Statement

The studies involving human participants were reviewed and approved by Ethics Committee of the Department of Psychology, UNIZG. The patients/participants provided their written informed consent to participate in this study.

## Author Contributions

IH: hypothesis, study design, data analysis, and drafting and writing the manuscript. AG: study design, data analysis, and writing the manuscript. GK: study design and recruitment of participants. All authors contributed to the article and approved the submitted version.

## Conflict of Interest

The authors declare that the research was conducted in the absence of any commercial or financial relationships that could be construed as a potential conflict of interest.

## References

[B1] Aarøe L. Petersen M. B. Arceneaux K. (2017). The behavioral immune system shapes political intuitions: why and how individual differences in disgust sensitivity underlie opposition to immigration. *Am. Political Sci. Rev.* 111 277–294. 10.1017/S0003055416000770

[B2] Ackerman J. M. Hill S. E. Murray D. R. (2018). The behavioral immune system: current concerns and future directions. *Soc. Personal. Psychol. Compass* 12:e12371. 10.1111/spc3.12371

[B3] Ackerman J. M. Tybur J. M. Blackwell A. D. (2020). What role does pathogen-avoidance psychology play in pandemics? *Trends Cogn. Sci.* 25 177–186. 10.1016/j.tics.2020.11.008 33293211 PMC7834713

[B4] Al-Shawaf L. Lewis D. M. G. Buss D. M. (2018). Sex differences in disgust: why are women more easily disgusted than men? *Emot. Rev.* 10 149–160. 10.1177/1754073917709940

[B5] Bačić N. (2007). Epidemije kuge na otoku Korčuli [The Plague Epidemics on Island Korčula]. *Hrvatska povijest i zdravlje* 4. Available online at: https://www.hzjz.hr/hrvatski-casopis-za-javno-zdravstvo/vol-3-broj-10-7-travnja-2007/

[B6] Baras F. (2020). Azijski bič tri puta harao Dalmacijom (3. dio): kolera na otocima [The Asian whip ravaged Dalmatia three times (part IIII: Cholera on the islands]. *Vijenac* 679. Available online at: https://www.matica.hr/vijenac/677/azijski-bic-tri-puta-harao-dalmacijom-29979/

[B7] Calvillo D. P. Ross B. J. Garcia R. J. B. Smelter T. J. Rutchick A. M. (2020). Political ideology predicts perceptions of the threat of COVID-19 (and susceptibility to fake news about It). *Soc. Psychol. Personal. Sci.* 11 1119–1128. 10.1177/1948550620940539

[B8] Cartaud A. Quesque F. Coello Y. (2020). Wearing a face mask against Covid-19 results in a reduction of social distancing. *PLoS One* 15:e0243023. 10.1371/journal.pone.0243023 33284812 PMC7721169

[B9] Corpuz R. D’Alessandro S. Adeyemo J. Jankowski N. Kandalaft K. (2020). Life history orientation predicts COVID-19 precautions and projected behaviors. *Front. Psychol.* 11:1857. 10.3389/fpsyg.2020.01857 32793087 PMC7393224

[B10] Curtis V. Biran A. (2001). Dirt, disgust, and disease. *Perspect. Biol. Med*. 44 17–31. 11253302 10.1353/pbm.2001.0001

[B11] Cvetnić Ž (2014). Kuga – bolest koja je promijenila svijet (I. dio) [Plague – the disease that changed the World (part I)]. *Veterinarska Stanica* 45 85–95.

[B12] Državno izborno povjerenstvo republike Hrvatske (DIP) (2020). *Izvješće o provedenim izborima za zastupnike u Hrvatski sabor 2020 [Report on conducted elections for Croatian parliament 2020]*. (DIP; State electoral commission of the Republic of Croatia). Available online at: https://www.izbori.hr/site/izbori-referendumi/izbori-za-zastupnike-u-hrvatski-sabor/67 (accessed March 2, 2021).

[B13] Duncan L. A. Schaller M. Park J. H. (2009). Perceived vulnerability to disease: development and validation of a 15-item self-report instrument. *Pers. Individ. Dif.* 47 541–546. 10.1016/j.paid.2009.05.001

[B14] Eterović I. (1994). Posebnosti zdravstvene zaštite na otocima [Specifics of health care on islands]. *Društ. Istraž* 4–5 467–485.

[B15] Everett J. A. C. (2013). The 12 item social and economic conservatism scale (SECS). *PLoS One* 8:e82131. 10.1371/journal.pone.0082131 24349200 PMC3859575

[B16] Falahi S. Kenarkoohi A. (2021). Sex and gender differences in the outcome of patients with COVID-19. *J. Med. Virol*. 93 151–152. 10.1002/jmv.26243 32603509 PMC7361270

[B17] Faulkner J. Schaller M. Park J. H. Duncan L. A. (2004). Evolved disease-avoidance mechanisms and contemporary xenophobic attitudes. *Group Process. Intergr. Relat.* 7 333–353. 10.1177/1368430204046142

[B18] Fenichel E. P. (2013). Economic considerations for social distancing and behavioral based policies during an epidemic. *Health Econ.* 32 440–451. 10.1016/j.jhealeco.2013.01.002 23419635 PMC3659402

[B19] Fincher C. L. Thornhil R. Murray D. R. Schaller M. (2008). Pathogen prevalence predicts human cross-cultural variability in individualism/collectivism. *Proc. Biol. Sci.* 275 1279–1285. 10.1098/rspb.2008.0094 18302996 PMC2602680

[B20] Goodall J. (1986). Social rejection, exclusion and shunning among the Gombe chimpanzees. *Ethol. Sociobiol.* 7 227–239. 10.1016/0162-3095(86)90050-6

[B21] Guthmiller J. J. Wilson P. C. (2020). Remembering seasonal coronaviruses. *Science* 370 1272–1273. 10.1126/science.abf4860 33303605

[B22] Hale T. Boby T. Angrist N. Cameron-Blake E. Hallas L. Kira B. (2020). *Variation in Government Responses to COVID-19: Version 9.0. Blavatnik School of Government Working Paper.* Available online at: www.bsg.ox.ac.uk/covidtracker (accessed March 2, 2021).

[B23] HINA (2020). U Zagrebu oštećeno više od 26000 grağevina, neuporabljivo ih je 1.900 [More than 26000 buildings damaged in Zagreb, 1.900 unusable]. N1. Available online at: https://www.tportal.hr/vijesti/clanak/u-zagrebu-osteceno-vise-od-26-000-gradevina-1-900-potpuno-neupotrebljivo-foto-20200328 (accessed December 27, 2020).

[B24] Iachini T. Coello Y. Frassinetti F. Senese V. P. Galante F. Ruggiero G. (2016). Peripersonal and interpersonal space in virtual and real environments: effects of gender and age. *J. Environ. Psychol.* 45 154–164.

[B25] IBM Corp (2019). *IBM SPSS Statistics for Windows, Version 26.0.* Armonk, NY: IBM Corp.

[B26] Jokic-Begic N. Lauri Korajlija A. Mikac U. (2020). Cyberhondria in the age of COVID-19. *PLoS One* 15:e0243704. 10.1371/journal.pone.0243704 33332400 PMC7746178

[B27] Jones B. C. Hahn A. C. Fisher C. I. Wang H. Kandrik M. Lee A. J. (2018). Hormonal correlates of pathogen disgust: testing the compensatory prophylaxis hypothesis. *Evol. Hum. Behav.* 39 166–169. 10.1016/j.evolhumbehav.2017.12.004

[B28] Karinen A. K. Molho C. Kupfer T. R. Tybur J. M. (2019). Disgust sensitivity and opposition to immigration: does contact avoidance or resistance to foreign norms explain the relationship? *J. Exp. Soc. Psychol* 84 1–11. 10.1016/j.jesp.2019.103817 [103817].,

[B29] Karwowski M. Kowai M. Groyecka A. Bialek M. Lebuda I. Sorokowska A. (2020). When in danger, turn right: does Covid-19 threat promote social conservatism and right-wing presidential candidates? *Hum. Ethol.* 35 37–48. 10.22330/he/35/037-048

[B30] Kim H. S. Sherman D. K. Updegraff J. A. (2016). Fear of ebola: the influence of collectivism on xenophobic threat reponses. *Psychol. Sci.* 27 935–944. 10.1177/0956797616642596 27207872

[B31] Kirinčić M. (2019). *Zdravstvena služba i zarazne bolesti na otoku Krku u vrijeme Prvoga svjetskog rata [Health service and infectious diseases on island Krkr during the WWI]. Otok Krk.* Available online at: https://otok-krk.org/krk/zdravstvena-slu%C5%BEba-i-zarazne-bolesti-na-otoku-krku-u-vrijeme-prvoga-svjetskog-rata (accessed March 3, 2021).

[B32] Kralj-Brassard R. (2016). Grad i kuga: dubrovnik 1691. godine [The town and the plague: dubrovnik in 1691.]. *Anali Zavoda za Povij. Znan. Hrvat. Akad. Znan. Umjet. Dubrovniku* 54 115–170.

[B33] Krams I. A. Jõers P. Luoto S. Trakimas G. Lietuvietis V. Krams R. (2021). The obesity paradox predicts the second wave of COVID-19 to be severe in western countries. *Int. J. Environ. Res. Public Health* 18:1029. 10.3390/ijerph18031029 33503828 PMC7908102

[B34] Krams I. A. Luoto S. Rantala M. J. Jõers P. Krama T. (2020). Covid-19: fat, obesity, inflammation, ethnicity, and sex differences. *Pathogens* 9:887. 10.3390/pathogens9110887 33114495 PMC7692736

[B35] Krebs D. (2008). Morality: an evolutionary account. *Perspect. Psychol. Sci.* 3 149–172. 10.1111/j.1745-6924.2008.00072.x 26158933

[B36] Lauri Korajlija A. Jokić-Begić N. (2020). COVID-19: concerns and behaviours in Croatia. *Br. J. Health Psychol.* 25 849–855. 10.1111/bjhp.12425 32779816 PMC7276766

[B37] Luoto S. Varella M. A. C. (2021). Pandemic leadership: sex differences and their evolutionary–developmental origins. *Front. Psychol.* 12:618. 10.3389/fpsyg.2021.633862 33815218 PMC8015803

[B38] MacMurray J. Comings D. E. Napolioni V. (2014). The gene-immune-behavioral pathway: gamma-interferon (IFN-γ) simultaneously coordinates susceptibility to infectious disease and harm avoidance behaviors. *Brain Behav. Immun.* 35 169–175. 10.1016/j.bbi.2013.09.012 24075848

[B39] Manson J. H. (2020). Right-wing authoritarianism, left-wing authoritarianism, and pandemic-mitigation authoritarianism. *Pers. Individ. Differ.* 167:110251. 10.1016/j.paid.2020.110251 32834284 PMC7365073

[B40] Markušić S. Stanko D. Korbar T. Belić N. Penava D. Kordić B. (2020). The Zagreb (Croatia) M5.5 Earthquake on 22 March 2020. *Geosciences* 10:252.

[B41] Mateus J. Grifoni A. Tarke A. Sidney J. Ramirez S. I. Dan J. M. (2020). Selective and cross-reactive SARS-CoV-2 T cell epitopes in unexposed humans. *Science* 370 89–94. 10.1126/science.abd3871 32753554 PMC7574914

[B42] Miłkowska K. Galbarczyk A. Mijas M. Jasienska G. (2021). Disgust sensitivity among women during the COVID-19 outbreak. *Front. Psychol.* 12:622634. 10.3389/fpsyg.2021.622634 33833715 PMC8021948

[B43] Miller S. L. Maner J. K. (2012). Overperceiving disease cues: the basic cognition of the behavioral immune system. *J. Pers.Soc. Psychol.* 102 1198–1213. 22329656 10.1037/a0027198

[B44] Mortensen C. R. Becker D. Ackerman J. M. Neuberg S. Kenrick D. (2010). Infection breeds reticence: the effects of disease salience on self-perceptions of personality and behavioral avoidance tendencies. *Psychol. Sci.* 21 440–447. 10.1177/0956797610361706 20424082

[B45] Mrakovčić M. Buršić E. (2017). “Društveni konzervatizam kao smjerokaz organizacije društvenog života [Social conservatism as a guideline for the organization of the social life]. [Conference presentation],” in *VI. Nacionalni sociološki kongres Hrvatskog sociološkog društva: Struktura i dinamika društvenih nejednakosti*, Croatia.

[B46] Murray D. R. Schaller M. (2010). Historical prevalence of infectious diseases within 230 geopolitical regions: a tool for investigating origins of culture. *J. Cross. Cult. Psychol.* 41 99–108. 10.1177/0022022109349510

[B47] Murray D. R. Prokosch M. L. Airington Z. (2019). PsychoBehavioroimmunology: connecting the behavioral immune system to its physiological foundations. *Front. Psychol.* 10:200. 10.3389/fpsyg.2019.00200 30804853 PMC6378957

[B48] Napolioni V. Murray D. R. Comings D. E. Peters W. R. Gade-Andavolu R. MacMurray J. (2014). Interaction between infectious diseases and personality traits: ACP1^∗^ C as a potential mediator. *Infect. Genet. Evol.* 26 267–273. 10.1016/j.meegid.2014.06.002 24933463

[B49] Navarrete C. D. Fessler D. M. T. (2006). Disease avoidance and ethnocentrism: the effects of disease vulnerability and disgust sensitivity on intergroup attitudes. *Evol. Hum. Behav.* 27 270–282. 10.1016/J.EVOLHUMBEHAV.2005.12.001

[B50] Navarrete C. D. Fessler D. M. Eng S. J. (2007). Elevated ethnocentrism in the first trimester of pregnancy. *Evol. Hum. Behav.* 28 60–66. 10.1016/j.evolhumbehav.2006.06.002

[B51] Oaten M. Stevenson R. J. Case T. I. (2009). Disgust as a disease-avoidance mechanism. *Psychol. Bull.* 135 303–321. 10.1037/a0014823 19254082

[B52] Ozdemir A. (2008). Shopping malls: measuring interpersonal distance under changing conditions and across cultures. *Field Methods* 20 226–248.

[B53] Prokosch M. L. Gassen J. Ackerman J. M. Hill S. E. (2019). Caution in the time of cholera: pathogen threats decrease risk tolerance. *Evol. Behav. Sci.* 13 311–334. 10.1037/ebs0000160

[B54] Raić Knežević A. (2020). *Prestrašeni Zagrepčani bježe na more. Policija zbog korone uvela kontrolne punktove na autocesti [Scared people of Zagreb running to the seaside: Police, due to corona, set up control points on the highway]. Telegram.* Available online at: https://www.telegram.hr/zivot/prestraseni-zagrepcani-bjeze-na-more-policija-uvela-kontrolne-punktove-na-autocesti-mostovi-za-otoke-su-zatvoreni/ (accessed December 27, 2020).

[B55] Rothbart M. K. Hanley D. Albert M. (1986). Gender differences in moral reasoning. *Sex Roles* 15 645–653. 10.1007/BF00288220

[B56] Rothman K. J. Greenland S. (1998). *Modern Epidemiology.* New York, NY: Lippincott Williams & Wilkins Publishers.

[B57] Rudan I. (2006). Health effects of human population isolation and admixture. *Croat. Med. J.* 47 526–531. 16909449 PMC2080449

[B58] Rudan I. Campbell H. Rudan P. (1999). Genetic epidemiological studies of eastern Adriatic island isolates, Croatia: objectives and strategies. *Coll. Antropol.* 23 531–546. 10646227

[B59] Rudan I. John V. Biloglav Z. Kujundzić-Tiljak M. Sonicki Z. Stevanović R. (2002). Epidemiološka obilježja zaraznih bolesti u izoliranim otočnim populacijama Republike Hrvatske [Epidemiological Characteristics of Infectious Diseases in Croatian Island Isolates]. *Lijec. Vjesn.* 124 70–74. 19702135

[B60] Sagar M. Reifler K. Rossi M. Miller N. S. Sinha P. White L. F. (2021). Recent endemic coronavirus infection is associated with less-severe COVID-19. *J. Clin. Invest.* 131:e143380. 10.1172/JCI143380 32997649 PMC7773342

[B61] Schaller M. (2006). Parasites, behavioral defenses, and the social psychological mechanisms through which cultures are evoked. *Psychol. Inq.* 17 96–101.

[B62] Schaller M. (2011). The behavioural immune system and the psychology of human sociality. *Philos. Trans. R. Soc. B* 366 3418–3426. 10.1098/rstb.2011.0029 22042918 PMC3189350

[B63] Schaller M. (2016). “The behavioral immune system,” in *The Handbook of Evolutionary Psychology*, ed. Buss D. M. (New Jersey, NJ: John Wiley & Sons, Inc), 206–224.

[B64] Schaller M. Murray D. R. (2008). Pathogens, personality, and culture: disease prevalence predicts worldwide variability in sociosexuality, extraversion and openness to experience. *J. Pers. Soc. Psychol.* 95 212–221. 10.1037/0022-3514.95.1.212 18605861

[B65] Schaller M. Murray D. R. (2010). “Infectious diseases and the evolution of cross-cultural differences,” in *Evolution, Culture, and the Human Mind*, eds Schaller M. Norenzayan A. Heine S. J. Yamagishi T. Kameda T. (New York, NY: Psychology Press), 243–256.

[B66] Schweon S. J. Edmonfs S. L. Kirk J. Rowland D. Y. Acosta C. (2013). Effectiveness of a comprehensive hand hygiene program for reduction of infection rates in a long-term care facility. *Am. J. Infect. Control* 41 39–44. 22750034 10.1016/j.ajic.2012.02.010

[B67] Skračić V. (2013). Otočani na malim otocima danas [Islanders on small islands today]. *Migracijske i Etničke Teme* 2 277–293. 10.11567/met.29.2.7

[B68] Sorokowska A. Sorokowski P. Hilpert P. Cantarero K. Frackowiak T. Ahmadi K. (2017). Preferred interpersonal distances: a global comparison. *J. Cross.Cult. Psychol.* 48 577–592. 10.1177/0022022117698039

[B69] Sorokowski P. Groyecka A. Kowal M. Sorokowska A. Bialek M. Lebuda I. (2020). Information about pandemic increases negative attitudes toward foreign groups: a case of COVID-19 outbreak. *Sustainability* 12:4912. 10.3390/su12124912

[B70] Stephan W. G. Ybarra O. Bachman G. (1999). Prejudice toward immigrants. *J. Appl. Soc. Psychol.* 29 2221–2237.

[B71] Stevenson R. J. Case T. I. Oaten M. J. (2009). Frequency and recency of infection and their relationship with disgust and contamination sensitivity. *Evol. Hum. Behav.* 30 363–368. 10.1016/j.evolhumbehav.2009.02.005

[B72] Stevenson R. J. Saluja S. Case T. I. (2021). The impact of the Covid-19 pandemic on disgust sensitivity. *Front. Psychol.* 11:600761. 10.3389/fpsyg.2020.600761 33551913 PMC7854913

[B73] Terrizzi J. A. Jr. Shook N. J. McDaniel M. A. (2013). The behavioral immune system and social conservatism: a meta-analysis. *Evol. Hum. Behav.* 34 99–108. 10.1016/j.evolhumbehav.2012.10.003

[B74] The jamovi project (2021). *jamovi* (Version 1.6) [Computer Software]. Available online at: https://www.jamovi.org (accessed March 1, 2021).

[B75] Tybur J. M. Inbar Y. Aarøe L. Barclay P. Barlow F. K. Barra M. D. (2016). Parasite stress and pathogen avoidance relate to distinct dimensions of political ideology across 30 nations. *Proc. Natl. Acad. Sci. U.S.A.* 113 12408–12413. 10.1073/pnas.1607398113 27791090 PMC5098626

[B76] Tybur J. M. Lieberman D. L. Griskevicius V. G. (2009). Microbes, mating, and morality: individual differences in three functional domains of disgust. *J. Pers. Soc. Psychol.* 29 103–122. 10.1037/a0015474 19586243

[B77] Varella M. A. C. Luoto S. da Silva Soares Varella Valentova J. (2021). COVID-19 pandemic on fire: evolved propensities for nocturnal activities as a liability against epidemiological control. *Front. Psychol*. 12:730. 10.3389/fpsyg.2021.646711 33828510 PMC8019933

[B78] Vitart V. Biloglav Z. Hayward C. Janicijevic B. Smolej-Narancic N. Barac L. (2006). 3000 years of solitude: extreme differentiation in the island isolates of Dalmatia, Croatia. *Eur. J. Hum. Genet.* 14 478–487. 10.1038/sj.ejhg.5201589 16493443

[B79] Vranić A. (2003). Personal space in physically abused children. *Environ. Behav.* 35 550–565.

[B80] Whittaker M. (2018). *Villages Wiped Out: Why Infectious Diseases Are so Much More Harmful to Isolated Peoples. The Conversation.* Available online at: https://theconversation.com/villages-wiped-out-why-infectious-diseases-are-so-much-more-harmful-to-isolated-peoples-91349 (accessed May 11, 2020).

[B81] Wu B. P. Chang L. (2012). *Pers. Individ. Diff.* 53 50–54. 10.1016/j.paid.2012.02.023Yong

[B82] Yong J. C. Choy B. K. C. (2021). Noncompliance with safety guidelines as a free-riding strategy: an evolutionary game-theoretic approach to cooperation during the COVID-19 pandemic. *Front. Psychol.* 12:729. 10.3389/fpsyg.2021.646892 33796057 PMC8008110

